# Effect of psychological intervention nursing on the emotion and quality of life of patients with recurrent spontaneous abortion

**DOI:** 10.12669/pjms.41.7.10661

**Published:** 2025-07

**Authors:** Chong Liu, Yanan Yang, Fengjiao Zhang, Qingmei Yang, Fang Yao, Lijuan Zhang

**Affiliations:** 1 Chong Liu, Department of Obstetrics and Gynecology, Hebei Research Institute for Family Planning Science and Technology, Shijiazhuang 050000, Hebei, China; 2 Yanan Yang, Department of Nursing, Hebei Research Institute for Family Planning Science and Technology, Shijiazhuang 050000, Hebei, China; 3 Fengjiao Zhang, Department of Obstetrics and Gynecology, Shijiazhuang Luquan District Maternal and Child Health Hospital, Shijiazhuang 050000, Hebei, China; 4 Qingmei Yang, Department of the 2^nd^ Obstetrics and Gynecology, Ningjin Maternal and Child Health Hospital, Xingtai 055550, Hebei, China; 5 Fang Yao, Department of Obstetrics and Gynecology, Shijiazhuang Luquan District Maternal and Child Health Hospital, Shijiazhuang 050000, Hebei, China; 6 Lijuan Zhang, Department of Pharmacy, Zhengding People’s Hospital, Shijiazhuang 050000, Hebei, China

**Keywords:** Emotion, Psychological intervention, Quality of life, Recurrent spontaneous abortion

## Abstract

**Objective:**

To explore the effect of psychological intervention nursing on the emotion and quality of life of patients with recurrent spontaneous abortion(RSA).

**Methods:**

This was a retrospective study. A total of 80 patients with RSA admitted to Hebei Research Institute for Family Planning Science and Technology from December 2022 to December 2023 were enrolled and randomly divided into two groups, with 40 cases in each group. Patients in the control group received traditional nursing, while those in the observation group were given additional psychological intervention measures on the basis of traditional nursing. Further statistical analysis was conducted to compare the anxiety, depression, self-efficacy, and quality of life of patients before and after nursing.

**Results:**

Before nursing, there was no significant difference in scores of all dimensions in Self-rating Anxiety Scale(SAS), Self-rating Depression Scale(SDS), General Self-Efficacy Scale(GSES), and SF-36 between the two groups (P>0.05). After nursing, SAS and SDS scores of both groups decreased than to those before nursing (P<0.05), both of which were significantly lower in the observation group than those in the control group (P<0.05). Meanwhile, scores of GSES and SF-36 were increased in both groups after nursing than those before nursing (P<0.05), with obviously higher scores in the observation group than those in the control group (P<0.05).

**Conclusion:**

The application of psychological intervention based on traditional nursing may significantly alleviate the anxiety and depression of patients with RSA, and improve their self-efficacy and quality of life.

## INTRODUCTION

Recurrent spontaneous abortion (RSA) is defined as two or more consecutive pregnancy loss before 28 weeks of gestation in China. However, there are differences in the criteria for determining the number of abortions and gestational time for RSA in different countries. The fact is that there are large number of patients around the world, and RSA troubles 1% to 3% of couples preparing for pregnancy according to statistics.^1^ RSA has a complicated pathogenesis and its specific mechanism remains unclear, which may be related to immune and inflammatory reactions^2^, uterine environment^3^, hormone changes^4^, maternal age^5^, and parental inheritance.^6^ So far, there is still a lack of effective therapies. Besides double whammy to patients’ physical and mental health, RSA may increase the risk of subsequent pregnancy, causing heavy economic and psychological burden on both patients and their families. As reported by previous studies, compared to those without RSA, women with RSA had significantly reduced physical function, general health, vitality, social function, emotional function, and mental health, as well as lower levels of happiness^7^; and it also had negative impact on the psychological condition of men, which might worsen with each consecutive abortion gradually.^8^

As such it highlights the necessity of providing systematic and thorough health knowledge education for RSA patients, and positive psychological intervention nursing based on patients’ conditions, so as to reduce the psychological pressure on patients and their families, and decrease the risk of pregnancy for patients eventually. Accordingly, this study was conducted by enrolling 80 patients with RSA in our hospital to investigate the impact of psychological intervention nursing on the emotion and quality of life of patients with RSA.

## METHODS

This was a retrospective study. Eighty patients with RSA admitted to Hebei Research Institute for Family Planning Science and Technology from December 2022 to December 2023. The enrolled patients were randomly divided into the control group and the observation group used the sealed envelope system, with 40 cases in each group.

### Ethical Approval:

The study was approved by the Institutional Ethics Committee of Hebei Research Institute for Family Planning Science and Technology (No.: 2023-121; Date: December 1, 2023), and written informed consent was obtained from all participants.

### Inclusion criteria:

- Patients aged 22-45 years old;
- Patients who had experienced ≥ two times of consecutive abortions spontaneously with the same partner;
- Patients with abortion within the gestational age of 12 weeks;
- Patients themselves and their families agreed to participate in this study and provided written informed consent.

### Exclusion criteria:

- Patients with severe mental illnesses (e.g., cognitive impairment);
- Patients with genetic diseases;
- Patients who did not cooperate with the survey.

### Methods

Patients in the control group and the observation group received nursing in two different ways. In the control group, patients were given traditional nursing, such as supervising patients to take medicine correctly and undergo physical examinations according to nursing regulations, strictly physical indicator monitoring, instructing patients to maintain personal hygiene and keep reasonable diet, etc.

Patients in the observation group were provided with psychological intervention on the basis of traditional nursing in the control group. The specific interventions are described as follows:

- Active communication with patients and their families frankly and sincerely to understand patients’ psychological state and concerns, and provide patients with a preliminary understanding of the medical team to gain the trust of patients and their families;
- Knowledge education on RSA and related examinations through on-site lectures or online science popularization to patients and their families to establish a correct understanding of RSA. It may be beneficial for alleviating negative emotions and mental stress that patients may experience spontaneously or passively due to the lack of scientific understanding, reducing their anxiety and sense of self-defense, improving their cooperation in examination and treatment, and thus fully mobilizing their subjective initiative;
- Personalized psychological interventions to alleviate negative emotions and psychological pressures experienced by different patients. A psychological intervention team was established to understand patients’ demands, guide them to relieve their emotions in a friendly and patient manner, and provide them with explanations, comfort, and guidance. Education and training would be given to family members as necessary to provide patients with emotional and practical support. The frequency of psychological counseling would be adjusted in a patient’s condition-dependent manner, supplemented by flexible psychological interventions (e.g., music and reading) to help patients establish a positive attitude towards life and enhance their confidence in active treatment. The two groups of patients were followed up for six months.

### Outcome measures:

Outcome measures included negative emotions, self-efficacy, and quality of life of the two groups of patients before and after intervention. To be specific, negative emotions of patients were evaluated by using Self-rating Anxiety Scale (SAS)^9^ and Self-rating Depression Scale (SDS).^10^ The total score was 80 points, and the specific score was directly proportional to the degree of anxiety and depression. Furthermore, the self-efficacy of patients was assessed with General Self-efficacy Scale (GSES).^11^ The total score was 40 points, and the score was also directly proportional to the level of self-efficacy. The 36 Item Short Form Health Survey (SF-36)^12^ was employed to evaluate the quality of life of patients from eight dimensions of physiological function, physiological function, energy, mental health, emotional function, general health, social function, and bodily pain. The total score for each dimension was 100 points, which was directly proportional to the level of patients’ quality of life.

### Statistical analysis:

Data processing and analysis in this study adopted SPSS 22.0 statistical software. Measurement data was expressed by (*χ̅*±*S*), and independent sample test was used for comparison between groups, inter-group comparison used t test. *P*<0.05 meant that the difference was statistically significant.

## RESULTS

In the control group, the average age, gravidity, and gestational age of RSA was (30.48±4.89) years old, (2.63±0.70) times, and (8.33±1.40) weeks, respectively. There were 23 cases with bachelor’s degree or above, 11 cases having college degree, and six cases of high school and junior high school degrees. Meanwhile, in the observation group, the average age, gravidity, and gestational age of RSA was (30.03±4.58) years old, (2.65±0.70) times, and (8.52±1.29) weeks, respectively. There were 21 cases with bachelor’s degree or above, 12 cases having college degree, and seven cases of high school and junior high school degrees. There was no significant difference in baseline data between the two groups, indicating comparability between groups.

In Table-I, there was no significant difference in SAS, SDS, and GSES scores between the control group and the observation group before nursing (*P*>0.05). After nursing, SAS and SDS scores of the two groups decreased compared to those before nursing (*P*<0.05), both of which were lower in the observation group than those in the control group, with statistically significant differences (*P*<0.05). Increased GSES scores were observed in both groups after nursing than those before nursing (*P*<0.05), with higher score in the observation group than that in the control group, showing a statistically significant difference (*P*<0.05).

**Table-I:** Assessment results of patients’ negative emotions and self-efficacy before and after nursing.

Items	Stages	Scores of the control group	Scores of the observation group	t	P
SAS	Before nursing	56.18±4.38	55.85±5.33	0.298	0.767
	After nursing	41.30±5.56*	33.65±3.76*	7.211	<0.001
SDS	Before nursing	55.88±4.87	54.93±4.70	0.887	0.378
	After nursing	43.70±5.70*	34.80±4.32*	7.874	<0.001
GSES	Before nursing	20.20±2.62	19.83±3.61	0.532	0.597
	After nursing	24.20±2.79*	27.18±3.03*	-4.566	<0.001

***Note:***

* *P*<0.05, significant difference in the score of the same item before and after nursing (the same below).

There was no significant difference in the comparison of physiological function, physiological function, energy, mental health, emotional function, general health, social function, and bodily pain between the control group and the observation group before nursing (*P*>0.05; Table-II). After nursing, scores of all eight dimensions in the control group and the observation group significantly increased compared to those before nursing (*P*<0.05), with higher scores in the latter group when compared with those in the former group, showing statistically significant differences (*P*<0.05).

**Table-II:** Assessment results of patients’ quality of life before and after nursing

Items	Stages	Scores of the control group	Scores of the observation group	t	P
Physiological function	Before nursing	58.95±4.78	59.23±3.77	-0.312	0.756
	After nursing	68.68±3.44*	71.50±3.69*	-3.545	0.001
Physiological function	Before nursing	61.65±4.15	60.94±3.45	0.82	0.415
	After nursing	66.90±3.10*	69.40±2.98*	-3.677	<0.001
Energy	Before nursing	43.20±4.84	42.13±4.14	1.067	0.289
	After nursing	51.20±3.25*	59.25±4.24*	-9.527	<0.001
Mental health	Before nursing	41.80±3.99	41.33±5.38	0.448	0.655
	After nursing	49.93±3.14*	53.28±3.46*	-4.531	<0.001
Emotional function	Before nursing	48.38±3.79	48.50±4.80	-0.129	0.898
	After nursing	58.65±3.41*	60.28±3.64*	-2.06	0.043
General health	Before nursing	53.33±3.93	53.05±4.10	0.306	0.76
	After nursing	60.50±3.25*	65.05±3.81*	-5.747	<0.001
Social function	Before nursing	61.18±5.42	62.86±3.38	-1.66	0.101
	After nursing	67.43±3.54*	70.23±3.29*	-3.669	<0.001
Bodily pain	Before nursing	66.88±5.51	67.63±6.67	-0.548	0.585
	After nursing	71.58±3.28*	74.43±3.82*	-3.578	0.001

## DISCUSSION

The results of this study showed that the scores of the three indicators of energy, mental health and emotional function before nursing were all lower than 50 points, which was lower than the scores of other indicators, indicating that recurrent abortion would have a negative impact on patients’ psychologically related quality of life. Many studies have shown that recurrent abortion seriously affects the quality of life of patients.^10^ A statistical study of Iranian patients with recurrent abortion showed significantly lower scores for physiological function, energy, mental health, emotional function, general health, and social function than healthy women,^7^ similar to the results presented here. It is necessary to increase targeted psychological intervention measures on the basis of traditional nursing to improve the mental and physical recovery of patients with recurrent abortion and improve the quality of life. In addition, after psychological intervention nursing, the scores of eight quality of life indicators of patients in this study increased significantly, and were significantly higher than those in the traditional nursing group. This shows that according to the specific situation of patients, providing them with health education, physical relaxation, psychological identification and other psychological intervention methods, and creating a calm and comfortable recovery environment for patients can alleviate bad emotions, improve happiness, and ultimately achieve the purpose of rapidly improving the quality of life. Iwanowicz-Palus G et al.^13^ believe that perceived available support in terms of emotion, instrumental and so on can help improve the quality of life of patients with recurrent abortion. Consistent with this research.

This study innovatively adopted the combination of deep communication, health education and psychological assistance to provide multi-faceted and multi-mode personalized psychological intervention means, and systematically verified that the mood and quality of life of RSA patients had a significant positive impact, and had certain guiding significance for the prognostic care of RSA patients. Patients with RSA are prone to having negative emotions. For instance, Fernlund A et al.^14^ included 177 patients with early abortion for surveying their psychological status, with 39.0% showing high-degree anxiety and 9.5% experiencing moderate or severe depressive symptoms. Painful experience of multiple abortions may increase patients’ anxiety and fear, which may rising the risk of adverse pregnancy outcomes (e.g., RSA) and forming a vicious cycle.^15^ Statistical analysis in this study showed that depression and anxiety were common in patients with RSA, which may be related to factors such as history of spontaneous abortion and therapeutic termination of pregnancy, number of live births, and duration of marriage.^16^ Meanwhile, some other researchers have also reported that women might experience significant post-traumatic stress, anxiety, and depression after early abortion, which would remain at a high level nine months after the abortion despite pain relief over time.^17^ Therefore, in view of patients’ personal situation, family, economic strength, educational background and many other aspects of the huge differences, the implementation of personalized psychological intervention to alleviate patients’ mental pressure and negative emotions is very important. No systematic validation has been found in other studies. Compared with traditional nursing, simultaneous psychological intervention could alleviate patients’ anxiety and depression, and improve their self-efficacy significantly. Therefore, appropriate psychological intervention measures can effectively alleviate the negative emotions of patients with RSA, enhance their self-regulation, self-decision-making and self-motivation abilities, and reconstruct their self-confidence to promote a rapid recovery. Similarly, Chang S et al.^18^ also discovered that empathetic care could significantly reduce stress and depression in women with RSA. In addition, the self-efficacy was inversely proportional to negative emotions such as anxiety and depression. As a result, psychological interventions to enhance patients’ sense of self-worth can reduce potential negative emotional impact of abortion on women, which was consistent with that concluded by Wang DN et al.^19^

### Limitations:

Owing to a potential selective bias of some patients, this study did not analyze the psychological status of pregnant women in the early stages of normal pregnancy. It is not yet clear whether the common symptoms of depression and anxiety in patients with RSA are attributed to the abortion or pregnancy pressure. Moreover, existing studies on this topic are mostly theoretical analyses, although psychological intervention for patients with RSA has been widely accepted by many healthcare professionals. In view of the above, further research is required to develop standardized and feasible interventional plans to provide technical support for clinical nursing of patients with RSA.

## CONCLUSIONS

To sum up, psychological intervention may significantly reduce patients’ anxiety and depression, and obviously improve their self-efficacy and quality of life compared with traditional nursing.

### Authors’ Contributions:

**CL** and **YY** carried out the studies, participated in collecting data, drafted the manuscript, are responsible and accountable for the accuracy or integrity of the work.

**FZ, QY, FY** and **LZ** performed the statistical analysis and participated in its design. Critical Review.

All authors have read and approved the final manuscript.
